# Supplementary figures and images for: Multi-targeting of K-Ras domains and mutations by peptide and small molecule inhibitors
Source: PLoS Comput Biol. 2022 Apr 26;18(4):e1009962. doi: 10.1371/journal.pcbi.1009962 (PMC9041843; doi:10.1371/journal.pcbi.1009962)

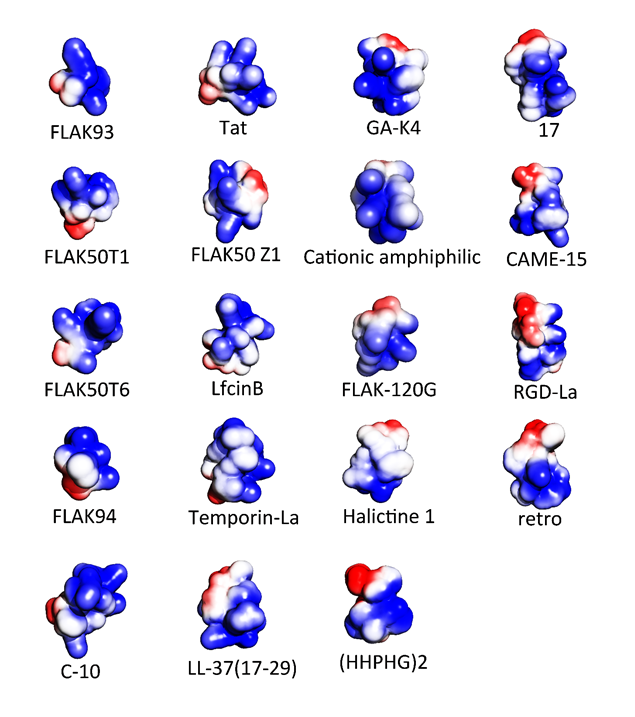

Supplement: S1 Fig — The positively-charged, negatively-charged and hydrophobic regions are shown in blue, red and gray colors, respectively. (TIF) [file pcbi.1009962.s001.tif]

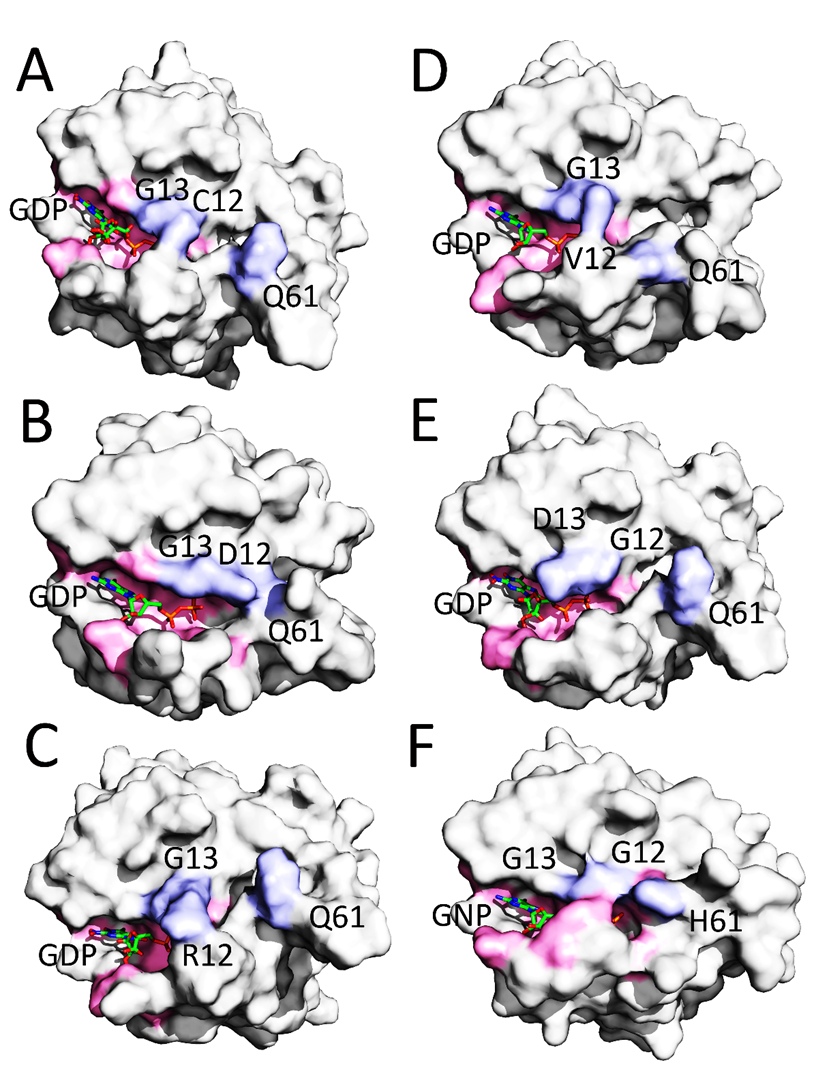

Supplement: S2 Fig — Surface representation of the structures of GDP-bound (A) K-RasG12C, (B) K-RasG12D, (C) K-RasG12R, (D) K-RasG12V, (E) K-RasG13D and (F) K-RasQ61H. The mutation sites and GTP/GDP-binding sites are shown in blue and magenta colors, respectively. (TIF) [file pcbi.1009962.s002.tif]

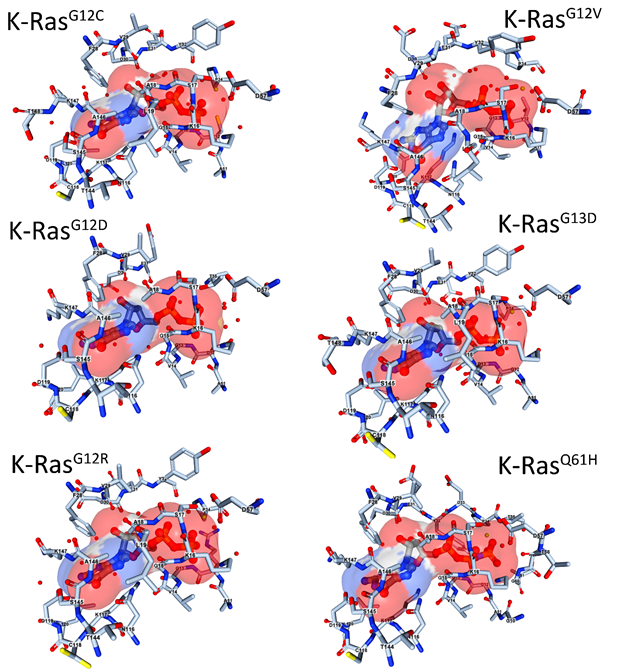

Supplement: S3 Fig — The surface electrostatic potential of GNP/GDP are shown in complex with K-Ras residues (sticks). The nucleotide atoms with positively-charged, negatively-charged and hydrophobic properties are shown in blue, red and gray colors, respectively. (TIF) [file pcbi.1009962.s003.tif]

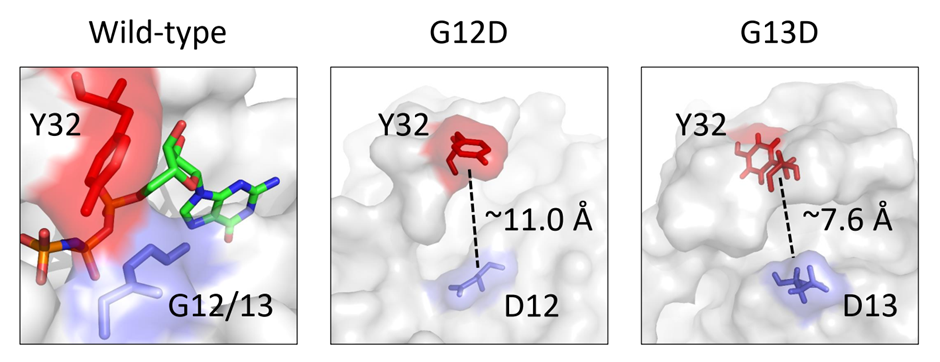

Supplement: S4 Fig — Interaction between Y32 and G12/13 in the wild-type K-Ras is also shown. (TIF) [file pcbi.1009962.s004.tif]

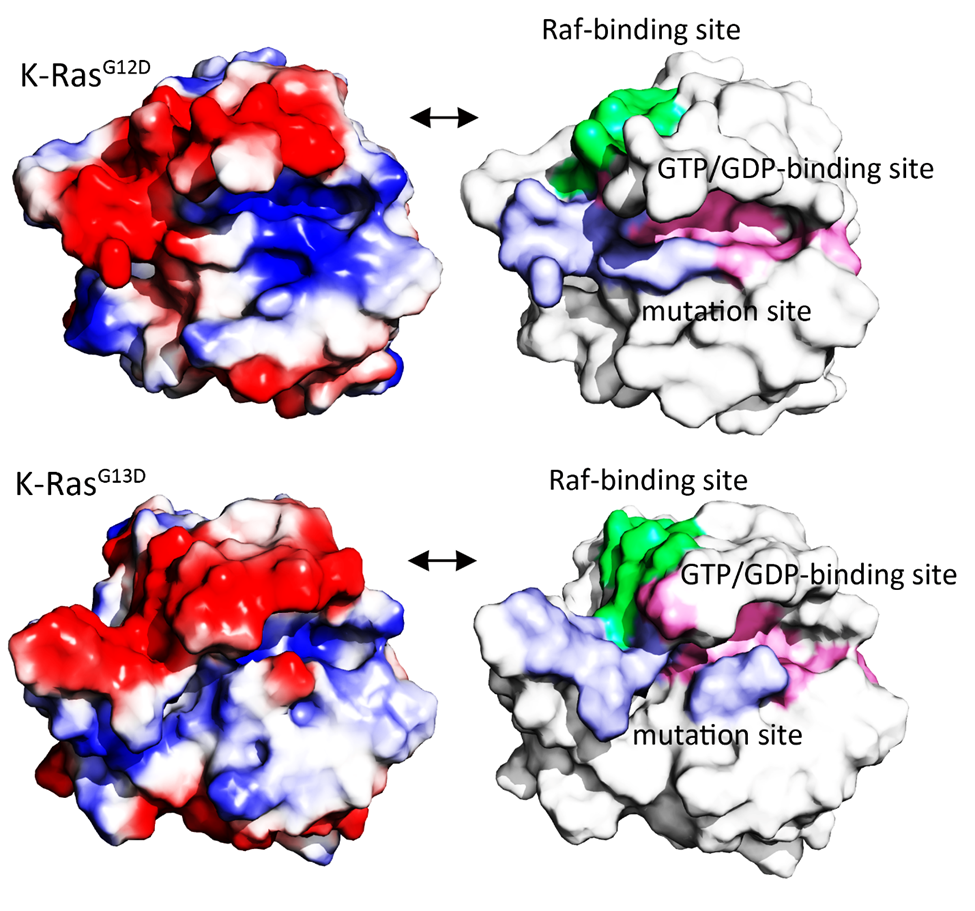

Supplement: S5 Fig — (Up) The surface representation of K-RasG12D and (down) K-RasG13D along with their surface electrostatic potentials. Red, blue and gray colors in the left sides refer to negatively-charged, positively-charged and hydrophobic regions of the proteins, respectively. The Raf-binding site, GTP/GDP-binding site and mutation site are shown in green, pink and blue colors, respectively, in the right side of Fig. (TIF) [file pcbi.1009962.s005.tif]

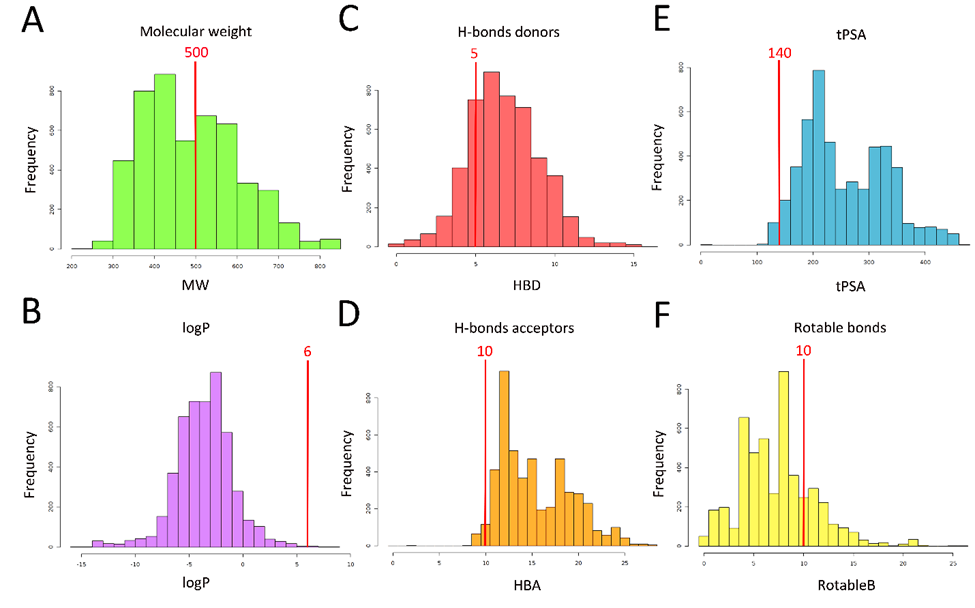

Supplement: S6 Fig — Distribution of (A) MW, (B) logP, (C) HBD, (D) HBA, (E) tPSA and (F) RotableB after FAFDrugs filtering based on Lipinski, Veber and Egan rules. Threshold of each parameter is shown with a red line. (TIF) [file pcbi.1009962.s006.tif]

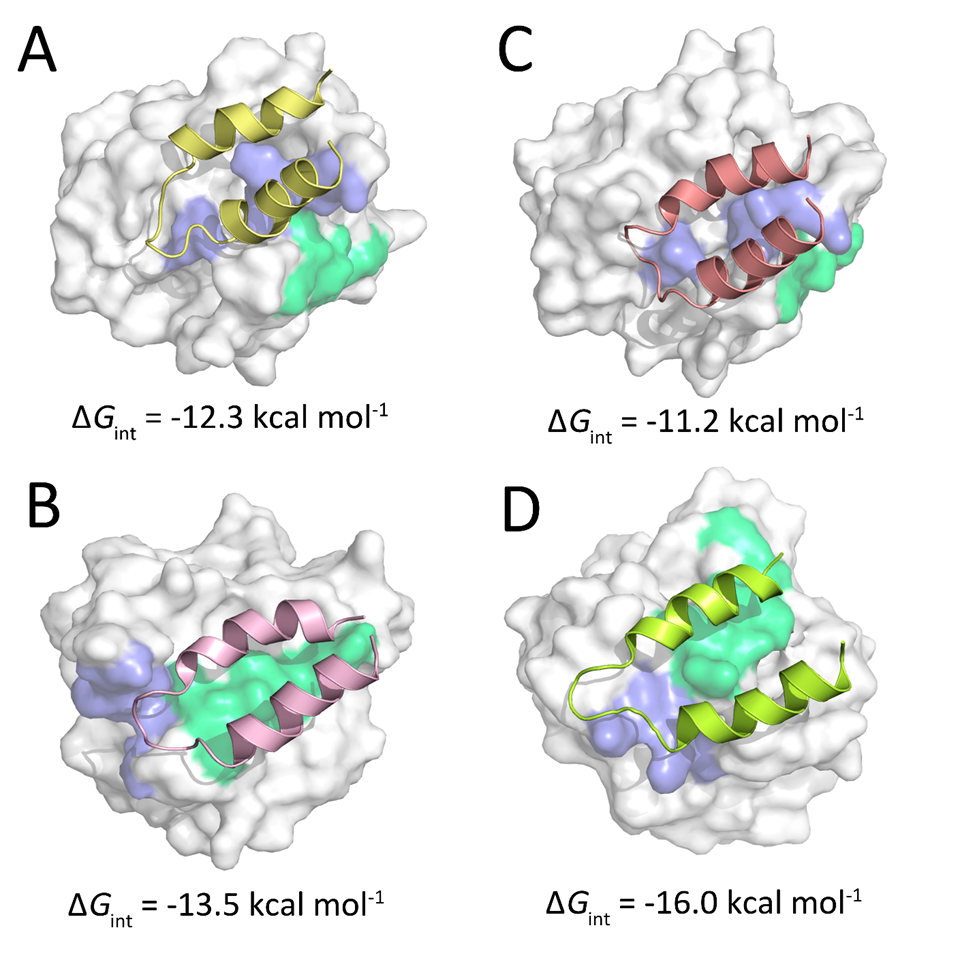

Supplement: S7 Fig — (A), (B), (C) and (D) refer to complex of the dimer peptide and K-RasG12C, K-RasG12R, K-RasG12V and K-RasQ61H. The Raf-binding and mutation sites of K-Ras are shown in green and blue colors, respectively. The binding energy of each peptide-protein complex are also provided. (TIF) [file pcbi.1009962.s007.tif]
