# Supplementary material for: Multi-targeting of K-Ras domains and mutations by peptide and small molecule inhibitors
Source: PLoS Comput Biol. 2022 Apr 26;18(4):e1009962. doi: 10.1371/journal.pcbi.1009962 (PMC9041843; doi:10.1371/journal.pcbi.1009962)
Supplement: S1 Table — Molecule as determined by MTT assay at different culture times. (DOCX) [file pcbi.1009962.s008.docx]

| Table S1. Percentage of AsPC1 cell death in the presence of 100 µM peptide. Molecule as determined by MTT assay at different culture times. | | | | |
| --- | --- | --- | --- | --- |
|  | 24h | 48h | 72h | 96h |
| 100 µM peptide: molecule | 22.5$\pm$1.2 | 31.8$\pm$3.3 | 48.6$\pm$2.6 | 36.2$\pm$3.1 |
