# Supplementary material for: Multi-targeting of K-Ras domains and mutations by peptide and small molecule inhibitors
Source: PLoS Comput Biol. 2022 Apr 26;18(4):e1009962. doi: 10.1371/journal.pcbi.1009962 (PMC9041843; doi:10.1371/journal.pcbi.1009962)
Supplement: S2 Table — (DOCX) [file pcbi.1009962.s009.docx]

| Table S2. Calculation of binding energy between peptides mutation site/Raf-binding site of K-Ras mutants. | | | | |
| --- | --- | --- | --- | --- |
| Peptide name | **G12C** | **G12R** | **G12V** | **Q61H** |
|  | **Δ*G_int_***  **(kcal.mol)** | **Δ*G_int_***  **(kcal.mol)** | **Δ*G_int_***  **(kcal.mol)** | **Δ*G_int_***  **(kcal.mol)** |
| FLAK93 | -6.3 | -7.2 | -6.9 | -7.3 |
| FLAK50T1 | -8.1 | -7.3 | -6.1 | -6.9 |
| FLAK50T6 | -7.3 | -6.9 | -6.8 | -8.1 |
| FLAK94 | -7.3 | -6.4 | -6.2 | -6.1 |
| C-10 | -9.3 | -7.3 | -8.1 | -6.8 |
| Tat | -10.1 | -7.1 | -6.8 | -8.9 |
| FLAK50 Z1 | -6.8 | -8.2 | -9.1 | -8.2 |
| LfcinB | -7.8 | -8.8 | -7.9 | -10.8 |
| Temporin-La | -7.1 | -8.3 | -9.1 | -7.9 |
| LL-37(17-29) | -7.1 | -6.9 | -7.9 | -8.1 |
| GA-K4 | -8.5 | -7.0 | -8.1 | -7.8 |
| Cationic Amphiphilic | -7.2 | -6.7 | -8.1 | -6.8 |
| FLAK-120G | -7.3 | -7.3 | -6.3 | -7.0 |
| Halictine 1 | -7.5 | -6.1 | -9.3 | -10.2 |
| (HHPHG)2 | -9.8 | -7.1 | -8.6 | -6.3 |
| 17 | -6.4 | -8.6 | -7.9 | -7.2 |
| CAME-15 | -7.3 | -10.9 | -7.1 | -7.7 |
| RGD-La | -7.4 | -8.0 | -8.8 | -9.8 |
| Retro | -8.9 | -7.2 | -9.2 | -7.6 |
