# Supplementary material for: Multi-targeting of K-Ras domains and mutations by peptide and small molecule inhibitors
Source: PLoS Comput Biol. 2022 Apr 26;18(4):e1009962. doi: 10.1371/journal.pcbi.1009962 (PMC9041843; doi:10.1371/journal.pcbi.1009962)
Supplement: S3 Table — (DOCX) [file pcbi.1009962.s010.docx]

**Table S3. The original and mutated sequence of LfcinB and Retro peptides.**

| Peptide name | Original sequence | Mutated sequence |
| --- | --- | --- |
| LfcinB | FK**C**RRWQWR**M**KK | FK**R**RRWQWR**R**KK |
| Retro | LG**G**IVSAVKKIV**D**FLG | LG**R**IVSAVKKIV**R**FLG |
