# Supplementary material for: Multi-targeting of K-Ras domains and mutations by peptide and small molecule inhibitors
Source: PLoS Comput Biol. 2022 Apr 26;18(4):e1009962. doi: 10.1371/journal.pcbi.1009962 (PMC9041843; doi:10.1371/journal.pcbi.1009962)
Supplement: S4 Table — (DOCX) [file pcbi.1009962.s011.docx]

| Table S4. Ten top-ranked compounds in case of their binding affinity to K-Ras^G12D^ | | | | | | | | |
| --- | --- | --- | --- | --- | --- | --- | --- | --- |
| Net charge | **RotableB** | **tPSA (Å^2^)** | **HBA** | **HBD** | **xlogP** | **MW (g mol^1^)** | **Binding energy (kcal mol^1^)** | **ZINC ID** |
| -3 | 6 | 241 | 15 | 4 | -2.53 | 424.179 | -10.12 | ZINC12360703 |
| -1 | 1 | 136 | 11 | 1 | -1.95 | 352.223 | -9.43 | ZINC13516349 |
| 0 | 5 | 149 | 10 | 5 | -0.40 | 325.325 | -9.37 | ZINC72481938 |
| -2 | 4 | 170 | 12 | 2 | -1.77 | 369.23 | -9.19 | ZINC31475037 |
| -2 | 4 | 170 | 12 | 2 | -1.77 | 369.23 | -9.03 | ZINC04533499 |
| -1 | 1 | 158 | 11 | 3 | -1.71 | 328.201 | -9.01 | ZINC12502230 |
| -1 | 1 | 158 | 11 | 3 | -2.32 | 328.201 | -8.93 | ZINC42854011 |
| -2 | 4 | 170 | 12 | 2 | -1.77 | 369.23 | -8.92 | ZINC04533500 |
| -2 | 4 | 149 | 11 | 1 | -1.81 | 353.231 | -8.86 | ZINC04533492 |
| 0 | 2 | 171 | 12 | 4 | -1.53 | 358.251 | -8.86 | ZINC08662656 |
