# Supplementary material for: Multi-targeting of K-Ras domains and mutations by peptide and small molecule inhibitors
Source: PLoS Comput Biol. 2022 Apr 26;18(4):e1009962. doi: 10.1371/journal.pcbi.1009962 (PMC9041843; doi:10.1371/journal.pcbi.1009962)
Supplement: Attachment — Submitted filename: Rebuttal letter.docx [file pcbi.1009962.s012.docx]

**24 October 2021**

**Plos Computational Biology**

**Dear Dr. MacKerell and Dr. Ben-Tal,**

Thank you very much for your letter of decision on the 21 Oct 2021 requesting a revision on our manuscript with ID number “**PCOMPBIOL-D-21-01581**”. We are very grateful to your and your reviewers' comments and suggestions. Based on the reviewers' precious comments, we have made careful modifications to the original manuscript according to these comments and addressed all the points brought up by the reviewers. We have highlighted all the changes made to the manuscript in response to the reviewers' comments.

Once again, we acknowledge your reviewers' comments very much, which are valuable in improving the quality of the manuscript.

Sincerely,

Ladan Teimoori-Toolabi

The corresponding author on behalf of all authors.

Here are our responses to the reviewer’s comments and list of corrections.

**Reviewer #1 (responses have been highlighted within the manuscript)**

**Reviewer #1: The manuscript by Mansour et al. “K-Raz targeting by peptide and molecule inhibitors” describes peptides and small molecules development as K-Ras inhibitors using different computational methods along with in-vitro study. The manuscript is well written and described high-quality research using advanced methodologies of molecular modelling and in-vitro assays. I recommend the article for publication in Plos Computational Biology after minor revisions.**

**1. It will be good to include some types of validation of the docking protocol in the manuscript.**

Thank you for your comment. We added some validations for the docking method and we added it in the text lines 390-396:

“First, we validated the docking method by a previously established structure of K-Ras in complex with an inhibitory peptide (ID: 5XCO). We re-generated the Kras-peptide complex by molecular docking and compared the results with the crystallography structure of this protein-peptide complex. There was a notable concordance between the docking model and crystallography structure in terms of binding interface and main residues involved in the H-bonding interactions (**Figure 3**).”

**2.** **The RMSD and RMSF graphs should be included in the manuscript to know about the stability and flexibility of the complex structure.**

Thank you for the precious comment. According to this comment, we added the RMSD and RMSF plots of K-Ras in the free and complex forms. Also we added this part and figure 6to the manuscript.

“To examine the dynamics and stability of K-Ras protein in the free and complex forms, we analyzed the Root Mean Square Deviation (RMSD) and Root Mean Square Fluctuation (RMSF) by MD trajectories resulting from a short MD simulation (10ns). Although the backbone RMSD of K-Ras in the complex form showed fluctuations at the beginning, the structure became stable at the last nanoseconds of the MD simulation. The variation of the RMSD graph was between 0 to 1.5Å (**Figure 6A**). To explore the impact of the designed peptide on the dynamics of the side-chain atoms, RMSF plots were also generated. In general, there were fewer fluctuations for the K-Ras residues in the complex form compared to the free protein, especially in the studied domains. This can be attributed to the local stability of K-Ras domains induced by the peptide. Interestingly, the dynamics of residues in the free K-Ras C-terminal was highly overlapped with the complex form (**Figure 6B**). This is compatible with the interaction mode of the peptide to K-Ras, which is bound to its target domains located in the N-terminal of the protein, but not the C-terminal. “

3**. The author should read the recently published article about K-ras by Mehreen et al, 2021, Journal of Biomolecular Structure and Dynamics, vol.38, 5488.**

Thank you for your suggestion. We read and used this article in our discussion section which is numbered 38.

4**. Throughout the manuscript small molecules should write instead of molecules**

We changed all “molecules” to “small molecules” throughout the manuscript.

**Reviewer #2** **(responses have been highlighted within the manuscript)**

**Reviewer #2: In the current work, the authors computationally screened peptide and small molecule databases to find peptide and small molecules targeting Kras. And based on the docking poses of top ranked peptides, they designed a peptide dimer. The inhibitory effect of the designed peptide dimer and molecule was assessed on the K-ras-positive pancreatic cancer cell lines. This work is novel, however, the current result is not fully supported by the presented data. The authors are recommended to consider the following comments to enhance the work.**

1. **The authors docked 19 peptides for Kras and found two top ranked peptide LfcinB and Retro targeting mutation site and Raf-binding site. While the LfcinB has more favorable docking score for mutation site over the Raf-binding site. The Retro has very similar docking scores for both sites. Only the Raf-binding site binding pose of Retro was considered as shown in Figure 3A and 3C. Did the author analyze the docking poses of Retro for both sites? For the peptide docking, did the author tried the global docking or only the mutation site and Raf-binding site being considered? Since the Kras protein size is quite trackable, MD simulations on the predicted peptide-protein complexes could help to further verify the docking poses.**

Thank you for your comment. We added these descriptions in the result section (line 527-538) according to this comment. Also the data of docking is given in Table 3:

“Both global and site-directed docking for K-Ras–peptide complexes were performed. In the global docking, a specific binding affinity was observed for the peptide to mutant K-Ras (Figure 5B). For site-directed docking, the Flex-pep dock algorithm was used and that analysis also confirmed the specific binding affinity of dimer peptide to the mutation and Raf-binding sites. However, two selected peptides had a high affinity for both mutation and Raf-binding sites. This was actually a good finding in our design since the main goal of designing a dimer peptide was to occupy both target sites, simultaneously. Therefore, it was great to see that two selected peptides have specific affinities to target domains, with higher affinity to one site but also high binding affinity to another site. So that they apparently can block the target sites more efficiently. PCA analysis proved the flexibility of each peptide around the linker sequence indicating that peptides could readily rotate around the linker. “

To have better insight about the dynamics of peptide-protein complex, we added the RMSD and RMSF plots generated from MD trajectories for both free K-Ras and K-Ras–peptide complex in figure 6 too.

**2. Affinity maturations on the two top ranked peptide were performed in the current work, however, no discussion was made. Figure 3 shows the overlaid docking poses of different designs of the two peptides. What are the mutations being made on the two peptides? How does such mutations help to improve the binding? More discussions are required here.**

Thank you for your comment. We added the sequences of original Retro/LfcinB and mutated peptides in the supplementary Table 2. We also discussed more about affinity maturation steps to clarify the approach as follows (lines 421-426):

“Among top-ranked mutant peptides based on the binding affinity calculations, the peptides with arginine-rich sites in N and/or C terminals were considered with high priority. This priority was due to the high cell-penetrating capability of arginine-rich peptides[40]. According to these criteria, we inserted two arginine residues in the Retro (G3→R3, D13→R13) and LfcinB (C3→R3, M10→R10) sequences (**Supplementary Table 2**).”

And this part (lines 457-460):

“Therefore, during affinity maturation steps, in addition to the peptide-Kras binding affinity, we also aimed to enhance the sequence similarity between the Retro and LfcinB peptides and previously established CPPs through the insertion of arginine residues to enhance their intracellular delivery.”

**3. Based on the predicted docking poses of the two top ranked peptides, the authors designed peptide dimer to utilize the binding benefits targeting both the mutation and Raf-binding sites. And they addressed that "we aimed to enhance the sequence similarity between the Retro-LfcinB dimer peptide and previously established CPPs to increase its intracellular delivery". Again, there is zero information about what mutations were conducted to modify the peptide dimer to enhance its profile. More information is required at here. And again, MD simulation on the predicted peptide-protein complex could help to verify the docking results.**

Thank you for your comment. We added these sentences to the manuscript according to this comment and some parts are summarized in Supplementary Table 2. We added peptide sequences and clarified the substitutions too. These sentences were added too (lines 421-426):

“Among top-ranked mutant peptides based on the binding affinity calculations, the peptides with arginine-rich sites in N and/or C terminals were considered with high priority. This priority was due to the high cell-penetrating capability of arginine-rich peptides[40]. According to these criteria, we inserted two arginine residues in the Retro (G3→R3, D13→R13) and LfcinB (C3→R3, M10→R10) sequences (**Supplementary Table 2**).”

And this part (lines 457-464):

“Therefore, during affinity maturation steps, in addition to the peptide-Kras binding affinity, we also aimed to enhance the sequence similarity between the Retro and LfcinB peptides and previously established CPPs through the insertion of arginine residues to enhance their intracellular delivery. Recently, arginine-rich peptides have also demonstrated stable interaction with mutant-type K-Ras resulting in the successful blockade of the K-Ras Raf-binding site [41]. However, only a few substitutions were performed to preserve the original properties of each peptide (**Supplementary Table 2**). "

Also, we added MD analyses to have better understanding about the dynamics of K-Ras–peptide complex (lines 539-552):

“To examine the dynamics and stability of K-Ras protein in the free and complex forms, we analyzed the Root Mean Square Deviation (RMSD) and Root Mean Square Fluctuation (RMSF) by MD trajectories resulting from a short MD simulation (10ns). Although the backbone RMSD of K-Ras in the complex form showed fluctuations at the beginning, the structure became stable at the last nanoseconds of the MD simulation. The variation of the RMSD graph was between 0 to 1.5Å (**Figure 6A**). To explore the impact of the designed peptide on the dynamics of the side-chain atoms, RMSF plots were also generated. In general, there were fewer fluctuations for the K-Ras residues in the complex form compared to the free protein, especially in the studied domains. This can be attributed to the local stability of K-Ras domains induced by the peptide. Interestingly, the dynamics of residues in the free K-Ras C-terminal was highly overlapped with the complex form (**Figure 6B**). This is compatible with the interaction mode of the peptide to K-Ras, which is bound to its target domains located in the N-terminal of the protein, but not the C-terminal.”

**4. The author mentioned "Molecular Dynamics (MD) trajectories were generated for both selected peptide-protein and molecule-protein complexes" in the Method section. However, the only discussion related to MD simulations is a PCA analysis on the peptide dimer to prove the flexibility of the linker. Does this done for MD trajectories on the peptide dimer alone or with protein? More analyses are required to fully support the design.**

Thank you. That is right. We should have had more discussions about the MD analyses. We did MD simulations for both free peptide (Figure 5B) and K-Ras–peptide complex (Figure 6). In the revised manuscript we discussed more about the dynamics properties of designed peptides using MD trajectories. These sentences were added according to this precious comment too (lines 539-552):

“To examine the dynamics and stability of K-Ras protein in the free and complex forms, we analyzed the Root Mean Square Deviation (RMSD) and Root Mean Square Fluctuation (RMSF) by MD trajectories resulting from a short MD simulation (10ns). Although the backbone RMSD of K-Ras in the complex form showed fluctuations at the beginning, the structure became stable at the last nanoseconds of the MD simulation. The variation of the RMSD graph was between 0 to 1.5Å (**Figure 6A**). To explore the impact of the designed peptide on the dynamics of the side-chain atoms, RMSF plots were also generated. In general, there were fewer fluctuations for the K-Ras residues in the complex form compared to the free protein, especially in the studied domains. This can be attributed to the local stability of K-Ras domains induced by the peptide. Interestingly, the dynamics of residues in the free K-Ras C-terminal was highly overlapped with the complex form (**Figure 6B**). This is compatible with the interaction mode of the peptide to K-Ras, which is bound to its target domains located in the N-terminal of the protein, but not the C-terminal. “

**5, There is actually a peptide inhibitor-Kras complex crystal structure available under PDB entry: 5XCOm (acsmedchemlett.7b00128). It is also an arginine rich peptide design. It changes the K-ras confirmation at the switch II. Is there any similarity between this design with the authors? Again, the authors should show their final peptide design. Is there any information can be borrowed from this crystal structure to interpret the current work?**

Thank you for the information and precious comment. According to this comment, we added this parts to the manuscript (lines 387-396).

” In order to select the candidate peptides, we screened the peptide library based on the binding affinity and interaction mode of peptides with K-Ras. To this end, molecular docking simulation is a reliable and rapid approach for the identification of peptides with a higher number of hydrogen bonds or other atom contacts with K-Ras [38]. First, we validated the docking method by a previously established structure of K-Ras in complex with an inhibitory peptide (ID: 5XCO). We re-generated the Kras-peptide complex by molecular docking and compared the results with the crystallography structure of this protein-peptide complex. There was a notable concordance between the docking model and crystallography structure in terms of binding interface and main residues involved in the H-bonding interactions (**Figure 3**).

Also in result and discussion part we added this part too (lines 461-469):

“Recently, arginine-rich peptides have also demonstrated stable interaction with mutant-type K-Ras resulting in the successful blockade of the K-Ras Raf-binding site [39]. However, only a few substitutions were performed to preserve the original properties of each peptide (**Supplementary Table 2**). Similar to our mutated LfcinB peptide, the peptide in the 5XCO crystallography file has Arginine-rich sequences in both N and C terminals[39]. This is actually a validation of our designed peptides, since during affinity maturation changes, we tried to increase the number of R/K residues in both N and C terminal of the peptides to enhance their cell-penetrating properties. Similar to our designed peptide, the peptide in the 5XCO binds to the K-Ras near to the switch domains.”

**6. Since the cell line inhibitory is not a direct binding assay, it is hard to tell how does the designed peptide dimer and the small molecule work together to bind K-ras. The authors can easily do another set of assay tests to verify the effects brought by the potential joint binding. Comparison between inhibitory effects brought by using the peptide dimer or molecule alone with the current inhibitory results using joint peptide:molecule can further elucidate the design of using both peptide and small molecule for Kras.**

Actually according to the lack of fund and sanctions we could not buy wild type and mutant K-ras proteins to see the effect of designed peptide dimer and the small molecules on them. We tried to buy them and in addition to the price, they would be transferred to us by next 6 months.
